# Supplementary material for: The 83 symptoms of tinnitus: Content overlap of commonly used scales for tinnitus burden
Source: PLoS One. 2026 Feb 9;21(2):e0340468. doi: 10.1371/journal.pone.0340468 (PMC12885300; doi:10.1371/journal.pone.0340468)
Supplement: Attachment — Submitted filename: Response to Reviewers.docx [file pone.0340468.s002.docx]

**Response to Reviewers**

***Reviewer #1:***

*The manuscript by Simões, J et al. presents a well-structured and clearly written study that constitutes a meaningful contribution to the field. The authors effectively address the important research question of content and overlap among tinnitus questionnaires assessing patient burden. This analysis offers a valuable step toward a consensus on PROMs selection and interpretation, thereby facilitating more direct and reliable comparisons between future studies, which is especially vital for clinical trial outcomes.*

Response: We would like to sincerely thank the reviewer for the time spent on our manuscript!

*Specific comments
It is important to note that all the selected questionnaires and subsequent analyses are conducted in English. This should be explicitly mentioned in the manuscript to ensure clarity for readers regarding the scope and applicability of the findings.*

Response: We totally agree with this valuable remark and included a corresponding note within the methods (line 113).

*Ln115 and 131: JPS or JS? I assume this refers to the same author (Simões, J.). The reference should be made consistent throughout the manuscript.*

Response: Thank you for reading carefully. We made the reference consistent.

*Ln 131: I suggest clarifying that this is the same team as in the first stage, in order to ensure transparency for the readers. Additionally, it would be helpful to explain the reason for this selection.*

Response: We hope we ensured more transparency by adding this information in line 130, thank you for the suggestion!

*Ln155: I strongly recommend citing R software as mentioned by them* [*https://intro2r.com/citing-r.html*](https://intro2r.com/citing-r.html)*.*

Response: Thank you for mentioning this! Very important remark to value their work! We cited accordingly.

*Conclusion: It may be important to include information about the overlap (even if limited) between the TFI and the THI, as these are the most widely used PROMs worldwide, both in research and clinical settings. The following key statement from the discussion could also be highlighted in this section as a take-away message: “A practical consequence of this finding is that tinnitus-burden PROMs should not be assumed to be interchangeable.”.*

Response: Thank you for pointing this out! We have added both points to the conclusion (lines 287–289).

*Overall, the comments are minor and should be straightforward to address.*

***Reviewer #2:***

*This is a rarely case of a manuscript, simple, elegant in design and perfectly delivered. My only suggestion is to improve the aesthetics of the tables, but this is only a fomating suggestion. But I would go forward to publication.*

Response: Thank you very much for taking the time to read our manucript and for your kind words! While we agree with your criticism of the tables' appearance, we would like to point out that they were formatted according to the journal's specifications.
